# Supplementary material for: Simultaneous Prediction, Determination, and Extraction of Four Polycyclic Aromatic Hydrocarbons in the Environment Using a UCON–NaH2PO4 Aqueous Two-Phase Extraction System Combined with High-Performance Liquid Chromatography-Ultraviolet Detection
Source: Molecules. 2022 Sep 30;27(19):6465. doi: 10.3390/molecules27196465 (PMC9571717; doi:10.3390/molecules27196465)
Supplement: Supplementary file 1 [file molecules-27-06465-s001.zip › molecules-1895797-supplementary.pdf]

Supplementary Material

$$Y_{11} = -49.21535 + 9.09988X_1 - 419.39680X_2 + 41.86507X_3 + 6.57000X_1X_2 - 0.20800X_1X_3 + 50.35000X_2X_3 - 0.15282X_1^2 - 0.74149X_2^2 - 8.00833X_3^2 \quad (7)$$

$$Y_{12} = 34.89227 - 1.22168X_1 - 63.96453X_2 - 0.97389X_3 + 0.39250X_1X_2 - 0.041750X_1X_3 + 3.88750X_2X_3 + 0.024247X_1^2 + 35.33872X_2^2 + 0.059938X_3^2 \quad (8)$$

$$Y_{21} = -42.94738 + 8.32950X_1 - 496.48838X_2 + 53.53507X_3 + 11.36500X_1X_2 + 0.040500X_1X_3 + 19.80000X_2X_3 - 0.19006X_1^2 + 56.76383X_2^2 - 8.99421X_3^2 \quad (9)$$

$$Y_{22} = 11.01348 - 0.35045X_1 - 7.33435X_2 - 0.53907X_3 - 1.71500X_1X_2 + 0.059000X_1X_3 - 7.10000X_2X_3 + 0.017294X_1^2 + 80.71105X_2^2 + 0.24673X_3^2 \quad (10)$$

$$Y_{31} = 102.08422 + 11.46552X_1 - 628.33280X_2 - 19.15722X_3 + 12.20000X_1X_2 + 0.53200X_1X_3 + 33.62500X_2X_3 - 0.29956X_1^2 + 105.10572X_2^2 - 1.64125X_3^2 \quad (11)$$

$$Y_{32} = 30.88023 - 1.12398X_1 - 41.55950X_2 - 1.53577X_3 + 0.87750X_1X_2 + 0.11375X_1X_3 - 0.43750X_2X_3 + 9.51566 \times 10^{-3}X_1^2 + 8.23281X_2^2 - 0.19875X_3^2 \quad (12)$$

$$Y_{41} = -71.25701 + 8.72601X_1 - 193.65779X_2 + 27.07282X_3 + 6.71500X_1X_2 + 0.15600X_1X_3 + 27.47500X_2X_3 - 0.17334X_1^2 - 143.60835X_2^2 - 5.97217X_3^2 \quad (13)$$

$$Y_{42} = 22.23053 - 0.87028X_1 - 22.91247X_2 - 1.37271X_3 - 0.030000X_1X_2 + 0.029500X_1X_3 - 1.60000X_2X_3 + 0.015959X_1^2 + 23.81168X_2^2 + 0.17978X_3^2 \quad (14)$$

where, Y_{ki} are the responses (k = 1 is PHE, k = 2 represents ANT, k = 3 is FLU, k = 4 represents PYR; i = 1 denotes extraction efficiency, i = 2 represents the enrichment factor.

Supplementary Table S1. Analysis of variance regression equation.

Object	Source	Sum of Squares	df	Mean Square	F Value	p-value Prob>F	
Y_{11}	Model	3329.45	9	369.94	66.7	< 0.0001	significant
	Residual	55.46	10	5.55			
	Lack of Fit	25.57	5	5.11	0.86	0.5659	not significant
	Pure Error	29.89	5	5.98			
	Cor Total	3384.91	19				
Y_{21}	Model	3521.15	9	391.24	65.28	< 0.0001	significant
	Residual	59.93	10	5.99			
	Lack of Fit	38.37	5	7.67	1.78	0.2713	not significant
	Pure Error	21.57	5	4.31			
	Cor Total	3581.08	19				
Y_{31}	Model	1904.3	9	211.59	68.31	< 0.0001	significant
	Residual	30.97	10	3.1			
	Lack of Fit	25.14	5	5.03	4.31	0.0674	not significant
	Pure Error	5.83	5	1.17			
	Cor Total	1935.27	19				
Y_{41}	Model	2261.23	9	251.25	67.05	< 0.0001	significant
	Residual	37.47	1	3.75			

			0				
	Lack of Fit	17.99	5	3.6	0.92	0.534	not significant
	Pure Error	19.49	5	3.9			
	Cor Total	2298.71	19				
Y_{12}	Model	38.48	9	4.28	54.49	< 0.0001	significant
	Residual	0.78	10	0.078			
	Lack of Fit	0.63	5	0.13	4.13	0.0728	not significant
	Pure Error	0.15	5	0.031			
	Cor Total	39.26	19				
Y_{22}	Model	44.42	9	4.94	69.26	< 0.0001	significant
	Residual	0.71	10	0.071			
	Lack of Fit	0.41	5	0.082	1.35	0.375	not significant
	Pure Error	0.3	5	0.061			
	Cor Total	45.13	19				
Y_{32}	Model	38.52	9	4.28	154	< 0.0001	significant
	Residual	0.28	10	0.028			
	Lack of Fit	0.21	5	0.042	3.18	0.1151	not significant
	Pure Error	0.067	5	0.013			

	Cor Total	38.8	19				
Y_{42}	Model	22.9	9	2.54	69.68	< 0.0001	significant
	Residual	0.37	10	0.037			
	Lack of Fit	5.72E-03	5	1.14E-03	0.016	0.9998	not significant
	Pure Error	0.36	5	0.072			
	Cor Total	23.27	19				

Y_{ki} is the responses(k=1 represents PHE,k=2 represents ANT,k=3 represents FLU,k=4 represents PYR;i=1 represents extraction efficiency,i=2 represents enrichment factor).

Supplementary Table S2. Recovery study and intra-day precision in real samples.

Sample	Object antibiotics	Spiked antibiotics (ng mL^{-1})	intraday precision (n=5)		intraday precision (n=7)	
			Recovery efficiency (E%)	RSD (%)	Recovery efficiency (E%)	RSD (%)
Water in Xiasantai River	PHE	0	ND		ND	
		10	97.52	0.23	98.01	1.62
		100	98.34	0.56	98.72	1.51
		500	98.28	1.28	97.97	1.36
		1000	98.16	0.41	97.62	1.17
	ANT	0	ND		ND	
		10	97.71	0.96	98.07	0.11
		100	98.29	1.53	99.14	1.02
		500	98.52	1.81	97.30	0.69
		1000	99.19	1.42	98.54	0.47
	FLU	0	ND		ND	
		10	98.18	2.14	98.26	2.31
		100	98.90	1.69	99.04	1.67
		500	98.27	1.58	98.47	1.28
		1000	97.71	1.31	97.96	1.23
	PYR	0	ND		ND	
		10	99.23	1.55	98.12	1.55
		100	98.01	0.93	98.21	0.45
		500	98.95	0.59	98.58	0.93
		1000	98.44	1.88	98.70	0.38
Water in Tashan Reservoir	PHE	0	ND		ND	
		10	98.69	1.26	97.20	1.87
		100	97.43	2.33	96.96	1.18
		500	98.54	1.59	96.12	0.67
		1000	98.13	1.16	98.48	0.12

	ANT	0	ND		ND	
		10	97.81	0.32	96.71	1.21
		100	97.46	1.81	97.71	0.26
		500	98.09	2.04	97.85	0.84
		1000	97.43	1.26	97.94	0.32
	FLU	0	ND		ND	
		10	96.76	1.48	98.43	1.88
		100	97.05	1.79	98.20	0.94
		500	97.89	0.28	97.66	1.70
		1000	98.32	1.93	97.53	1.43
	PYR	0	ND		ND	
		10	98.62	1.69	97.95	1.15
		100	98.39	1.52	99.30	0.11
		500	98.42	1.17	99.11	0.75
		1000	97.52	0.83	98.82	1.78
	PHE	0	ND		ND	
		10	99.25	1.03	98.09	0.12
		100	98.86	0.68	98.70	1.10
		500	98.75	1.13	99.23	0.61
		1000	98.92	1.48	99.22	1.73
	ANT	0	ND		ND	
		10	97.94	1.91	98.34	1.27
Topsoil		100	98.52	0.77	98.19	0.24
		500	99.61	1.37	98.30	1.79
		1000	99.26	1.82	98.77	2.11
	FLU	0	ND		ND	
		10	97.72	0.73	97.67	1.40
		100	99.51	0.67	99.13	0.98
		500	98.93	1.19	98.02	1.59

		1000	98.50	0.91	98.44	1.41
	PYR	0	ND		ND	
		10	99.07	1.01	98.49	1.18
		100	98.22	1.39	98.27	0.58
		500	97.62	0.42	97.56	0.87
		1000	98.30	1.29	98.07	0.62
Subsoil	PHE	0	ND		ND	
		10	97.78	2.07	97.94	1.89
		100	98.39	1.44	99.07	1.47
		500	97.47	1.89	97.54	0.24
		1000	98.44	0.71	98.41	2.45
	ANT	0	ND		ND	
		10	99.21	0.92	99.84	1.52
		100	98.70	1.21	98.62	0.51
		500	97.76	0.23	97.34	1.05
		1000	98.73	1.74	98.11	0.47
	FLU	0	ND		ND	
		10	97.68	0.91	98.50	1.24
		100	98.95	1.54	97.79	0.67
		500	98.50	0.48	98.66	1.69
		1000	98.37	1.65	97.45	1.13
	PYR	0	ND		ND	
		10	99.23	1.14	98.41	0.72
		100	98.83	1.81	97.76	1.38
		500	98.04	0.58	98.65	0.58
		1000	97.71	1.33	98.22	1.42

ND: not found.

Supplementary Table S3. Recovery study and intra-day precision in real samples.

Sample	Object antibiotics	Spiked antibiotics (pg mL^{-1})	intraday precision (n=5)		intraday precision (n=7)	
			Recovery efficiency (E%)	RSD (%)	Recovery efficiency (E%)	RSD (%)
Water in Xiasantai River	SDZ	0	ND		ND	
		10	96.22	1.19	97.10	0.92
		100	97.53	1.59	98.15	2.06
		500	98.27	1.94	98.79	1.03
		1000	98.31	1.37	99.01	1.25
	SMT	0	ND		ND	
		10	97.44	3.06	97.55	2.51
		100	98.50	2.24	98.27	1.29
		500	96.68	1.73	97.64	2.28
		1000	97.91	1.67	97.08	1.93
Water in Tashan Reservoir	SDZ	0	ND		ND	
		10	97.63	2.50	99.40	1.32
		100	98.40	1.60	97.66	2.06
		500	97.84	0.94	97.89	2.42
		1000	97.33	1.23	98.55	1.91
	SMT	0	ND		ND	
		10	97.99	1.22	98.06	2.26
		100	97.76	1.40	96.81	2.04
		500	97.79	0.97	97.91	1.59
		1000	98.27	0.69	97.50	1.80
Milk	SDZ	0	ND		ND	

		10	97.49	2.08	96.14	1.64
		100	97.58	0.66	96.43	0.41
		500	97.95	1.28	97.26	1.16
		1000	98.07	0.57	97.69	0.49
	SMT	0	ND		ND	
		10	96.58	1.56	98.59	2.84
		100	96.34	1.34	97.38	2.26
		500	96.71	1.05	98.32	2.12
		1000	97.85	0.74	97.81	1.77
Egg	SDZ	0	ND		ND	
		10	97.18	1.99	97.32	0.49
		100	96.84	2.39	98.66	2.42
		500	97.46	0.44	98.47	2.71
		1000	96.81	2.57	97.59	1.71
	SMT	0	ND		ND	
		10	97.80	2.08	96.09	1.71
		100	97.57	1.28	97.08	3.09
		500	97.03	0.84	97.22	2.13
		1000	96.90	2.51	97.31	1.58

ND: not found.
